# The association between physical stature and myopia in elementary and junior high school graduates in Chongqing, China

**DOI:** 10.3389/fmed.2025.1530960

**Published:** 2025-06-02

**Authors:** Jing Zhang, Ruili Li, Yong Zhang, Wensheng Tang, Dan Ao, Li He, Kun Yang, Xiaoya Qi, Xiyuan Zhou

**Affiliations:** ^1^Department of Health Management Center, The First Affiliated Hospital of Chongqing Medical University, Chongqing, China; ^2^Department of Special Medical Service Centre, The First Affiliated Hospital of Chongqing Medical University, Chongqing, China; ^3^School of Public Health, Chongqing Medical University, Chongqing, China; ^4^Health Medicine Center, The Second Affiliated Hospital of Chongqing Medical University, Chongqing, China; ^5^Department of Ophthalmology, The Second Affiliated Hospital of Chongqing Medical University, Chongqing, China

**Keywords:** myopia, physical stature, height, spherical equivalent refraction, children

## Abstract

**Purpose:**

This study aims to investigate the relationship between myopia and physical statures in elementary and junior high school graduates.

**Methods:**

Eight hundred and seventy-one elementary graduates and 752 junior high graduates in the urban area of Chongqing in China were recruited when they came to the hospital for their physical examination at enrollment. All the participants underwent anthropometric measurements, ocular examination, including visual acuity, slit lamp, non-cycloplegic refraction, and a questionnaire survey on demographics and life habits related to eye use. Univariate and Multivariate regression was used to analyze the relationships between physiques and SER (spherical equivalent refraction).

**Results:**

The mean ages were 12 ± 0.5 years and 15 ± 0.5 years for graduates in elementary school and in junior high school, respectively, the number of myopic children were 73.8% (643/871) and 82.6% (621/752) in elementary school and in junior high school, respectively. The regression coefficients between SER and height for elementary school graduates were significant in the right eye (*B* = −0.002, *p* = 0.011) and in the left eye (*B* = −0.025, *p* = 0.005) by univariate analysis. These negative associations between SER and height in elementary graduates were significant after adjusting for multiple covariates (*B* = −0.020, *p* = 0.025 for right eye and B = −0.022, *p* = 0.014 for left eye). On the contrary, physical indexes were not significantly related to SER in junior high school graduates in univariate analysis and multivariate analysis.

**Conclusion:**

Accelerated height growth at elementary school may increase the risk of myopia by accelerating the process of emmetropization, but this relationship may be covered up by other risk factors, especially the increased near-sight activity due to increased academic work at higher grades. Further study is warranted to explore this relationship in children of different ages.

## Introduction

Myopia is one of the major causes of poor vision in children and adolescents (1). By 2050, 4.7 billion people will have myopia, and 938 million people will have severe myopia, accounting for approximately 50 and 10% of the global population, respectively (2). In general, the highest prevalence of myopia exists in East Asia, highest among China (3, 4). The prevalence of myopia among young Chinese keeps rising annually, with around 80–90% of myopia at the end of 12 years of school (5). Myopia can increase the risk of ocular complications, such as cataracts, glaucoma, retinal detachment, and macular degeneration (6). As far as students are concerned, myopia affects school performance and limits employability. It impairs an individual’s quality of life (7, 8). Due to the related ocular healthcare and vision impairment, the economic costs of myopia are significant (9, 10).

Factors such as extended time near-sight work, insufficient outdoor activities, academic pressure, and ethnic and family history of high myopia have been proven to be associated with myopia (11–13). Besides, physical development was also thought to be related to myopia because taller and overweight people tend to have a large eye and longer axis length, which may lead to myopia (14, 15). However, the association between physical growth and refractive error is still controversial. In Asian children, a greater prevalence of myopia in children with higher height has been reported (14, 16, 17). Tao et al. (18) found that both height and the changes in height were negatively correlated to SER in 414 Chinese children aged 6–9 years in 5 years followed up. Kearney et al. (10) also reported that persistent myopes (≤ − 0.5D) were significantly taller than persistent emmetropes (−0.5D to +1.0D) in 5 to 20-year-old participants. On the contrary, Saw’s study showed no significant relation between height and myopia (19).

In a study on 19-year-old male conscripts in South Korea, myopia prevalence was not associated with height (*p* = 0.159), weight (*p* = 0.571), or body mass index (*p* = 0.323) (20). An Israeli study on participants aged 17 to 19 also reported that myopia was not related to body stature (21). Another study reported that refractive error or axis length in 11- and 15-year-old children was unrelated to height (22). The findings in previous studies may be ascribed to the differences in participant’s age and living conditions. Meanwhile, most myopia starts in young schoolchildren and progresses in early adolescence (23). Therefore, it is interesting to study the effect of physical development on refraction development in young children.

Previously, we investigated the association between physical stature and myopia in first-year students at the university and found that the association was insignificant in those young adults who had high academic achievement and were admitted to prestigious universities (24). We hypothesized that increased near-sight activity may attenuate the relationship between body size and myopia. But we could not measure the spherical equivalent refraction, which would be more objective and sensitive than diagnosing myopia by vision acuity chart in the school setting. Younger children were included in this study to capture the association between physical stature and myopia.

This study aimed to find out whether physical stature growth influences the spherical equivalent refraction (SER) in younger children and provide evidence that will contribute to the understanding of the development of myopia and the seeking of interventions for school-aged children in the future.

## Methods

### Study participants

In this cross-sectional study, the subjects were Chongqing’s elementary and junior high school graduates. The Ethics Committee of Chongqing Medical University obtained ethical approval (No. CAF52704054B). The purpose and content of the study were explained to all subjects and their parents or legal guardians, and written consent was obtained from all subjects involved (the participants’ legal guardians/next of kin).

All elementary school graduates aged 11–13 and junior high school graduates aged 14–16 who came to The Second Affiliated Hospital of Chongqing Medical University for their enrollment physical examination were recruited. Students who refused to participate or had a medical history of eye diseases such as strabismus, amblyopia, high astigmatism (astigmatism more significant than two diopters or anisometropia greater than two diopters), ocular inflammation, ocular trauma, corneal disease, congenital cataract, choroid or retinal disorders were excluded from the study.

### Ocular examinations

Ocular examinations containing visual acuity, slit-lamp examination, direct ophthalmoscopy, and non-cycloplegic refraction. All subjects underwent measurement of uncorrected distance visual acuity (UCDVA) at 5 m (standard logarithmic visual acuity E chart) and recorded in logMAR scores. Visual acuity was tested with and without refractive correction for those wearing spectacles. An auto refractometer (HRK-7000A, Huvitz Co. Ltd.) was used to measure non-cycloplegic refraction in a darkened room. Some previous studies have showed that there’s no difference in accurate refraction measured with or without cycloplegic in in children beyond 10 years old (4, 9). Each eye of each student was measured at least thrice. If the difference of refractive error between measurements reached 0.50 diopters (D) or above, an additional measurement were added. Mean values of multiple tests were used for analysis.

### Physical examination

Physicians from the Department of Health Medical Center conducted other conventional physical examinations, including height (centimeters), weight (kilograms), and blood pressure (mmHg). Basic information such as age and sex were also recorded. Examiners had basic training to reduce inter observer bias.

### Questionnaire survey

A self-administered, web-based questionnaire collected demographic, sociological, and behavioral information about eyesight: to reduce individual errors, the same investigating team administered all survey questionnaires.

### Definitions

The spherical equivalent refraction (SER) was converted by adding the spherical refraction and half the cylindrical refraction. Myopia was defined as SER < = −0.50 D in either eye. The myopia was divided into three levels according to SER: low myopia −3.0 D < SER < −0.5 D; moderate myopia −6.0 D < SER ≤ −3.0 D; high myopia SER ≤ −6.00D (25).

Overweight and obesity were defined according to China’s national comprehensive evaluation standards of children and adolescent development. For elementary school graduates, the body mass index for overweight is 21.0–24.7 and 21.9–24.5 for males and females, respectively, and the body mass index for obesity is > = 24.7 and > = 24.5 for males and females, respectively. For junior high school graduates, the body mass index for overweight is 23.1–26.9 and 23.4–26.9 for males and females, respectively, and the body mass index for obesity is > = 26.9 for both sexes (26).

### Quality control

Before the study started, every research team member, including two experienced ophthalmologists, two qualified optometrists, and three postgraduates, was trained. All instruments were checked and adjusted before the examination.

### Data analysis

Categorical and continuous variables were summarized as proportions or mean± standard deviation and compared between subjects with and without myopia using the chi-square test or the analysis of variance. The association between lifestyle factors and the occurrence of myopia was analyzed using univariate and multivariate linear regression. All statistical analysis was conducted using the software SPSS 19.0.

## Results

### Characteristics of the study participants

Of the 1806 eligible students, 183 participants were excluded due to missing data and excluded criteria. Finally, a total of 1,623 students were included in the analysis. The correlation coefficient of SER between left and right eye was 0.869 (*p* < 0.001). The percentage of myopia in elementary school graduates was 73.8% (643/871), and the percentage of myopia in junior high school graduates was 82.6% (621/752) among who visited hospital for examination. Only 26.2 and 17.4% had emmetropia in elementary and junior high graduates, respectively. The details of subjects’ characteristics, including demographics, physique measurements, ocular examinations, and learning and living style, were compared by gender and shown in Table 1.

**Table 1:** Characteristics of subjects (*N* = 1,623).

Variable	ESG		JSG		P1	P2
	Male(*N* = 451)	Female(*N* = 420)	Male(*N* = 387)	Female(*N* = 365)		
Age (year)	12.12 ± 0.98	11.95 ± 0.68	15.02 ± 0.94	14.93 ± 0.77	0.002	0.133
Height (cm)	159.24 ± 8.42	157.90 ± 5.69	172.53 ± 6.63	161.42 ± 6.16	0.004	0.001
Weight (Kg)	49.47 ± 12.08	48.10 ± 9.79	64.91 ± 13.88	53.10 ± 9.17	0.053	0.001
Body mass index	19.34 ± 3.64	19.23 ± 3.36	21.79 ± 4.51	20.40 ± 3.56	0.633	0.001
Obesity					0.001	0.001
None (n, %)	310(68.7)	339(80.7)	255(65.9)	311(85.2)		
Overweight (n, %)	100(22.2)	50(11.9)	76(19.6)	38(10.4)		
Obesity (n, %)	41(9.1%)	31(7.4)	56(14.5)	16(4.4)		
SBP (mmHg)	111.29 ± 11.86	109.03 ± 10.78	117.60 ± 11.97	111.41 ± 11.09	0.002	0.001
DBP (mmHg)	64.92 ± 7.07	65.76 ± 7.66	66.85 ± 7.91	67.67 ± 8.20	0.079	0.141
Log MAR-right eye	0.41 ± 0.38	0.47 ± 0.37	0.57 ± 0.41	0.59 ± 0.38	0.011	0.539
Log MAR-left eye	0.40 ± 0.39	0.43 ± 0.36	0.54 ± 0.41	0.54 ± 0.40	0.214	0.937
SER-Right eye	−2.19 ± 2.02	−2.20 ± 1.78	−3.22 ± 2.29	−3.03 ± 2.02	0.936	0.239
SER-Left eye	−1.95 ± 2.05	−2.00 ± 1.85	−2.93 ± 2.38	−2.70 ± 2.15	0.688	0.172
Myopia (right eye)					0.014	0.075
Negative (n, %)	134(29.7)	94(22.4)	72(18.6)	59(16.2)		
Low (n, %)	168(37.3)	190(45.2)	109(28.2)	120(32.9)		
Middle (n, %)	129(28.6)	126(30.0)	157(40.6)	158(43.3)		
High (n, %)	20(4.4)	10(2.4)	49(12.7)	28(7.7)		
Without sibling					0.733	0.028
Yes (n, %)	203(45.0)	184(43.8)	192(49.6)	151(41.4)		
Parents myopia					0.495	0.094
Father (n, %)	81(18.0)	73(17.4)	73(18.9)	60(16.4)		
Mother (n, %)	91(20.2)	106(25.2)	72(18.6)	72(19.7)		
Both (n, %)	100(22.2)	74(17.6)	83(21.4)	57(15.6)		
Mother education					0.382	0.001
Low (n, %)	203(45.0)	180(43.0)	191(49.4)	224(61.5)		
Middle (n, %)	230(51.0)	214(51.1)	178(46.0)	133(36.5)		
High (n, %)	18(4.0)	25(6.0)	18(4.7)	7(1.9)		
Father education					0.276	0.105
Low (n, %)	200(44.3)	180(43.0)	195(50.4)	202(55.5)		
Middle (n, %)	224(49.7)	202(48.2)	162(41.9)	146(40.1)		
High (n, %)	27(6.0)	37(8.8)	30(7.8)	16(4.4)		
Academic achievement					0.020	0.044
Low (n, %)	24(5.3)	39(9.3)	73(18.9)	86(23.6)		
Middle (n, %)	264(58.5)	257(61.2)	170(43.9)	173(47.4)		
High (n, %)	163(36.1)	124(29.5)	144(37.2)	106(29.0)		
Homework					0.155	0.757
<1 h (n, %)	102(22.6)	73(17.4)	31(8.0)	22(6.0)		
1 ~ 2 h (n, %)	217(48.1)	200(47.6)	96(24.8)	90(24.7)		
2 ~ 3 h (n, %)	100(22.2)	110(26.2)	115(29.7)	113(31.0)		
>3 h (n, %)	32(7.1)	37(8.8)	145(37.5)	140(38.4)		
Take a break in eyes using					0.686	0.933
Seldom (n, %)	98(21.7)	84(20.0)	101(26.1)	95(25.0)		
Sometime (n, %)	268(59.4)	261(62.3)	236(61.0)	226(61.9)		
Often (n, %)	85(18.8)	74(17.7)	50(12.9)	44(12.1)		
Attending out-school course						
Yes (n, %)	391(86.7)	374(89.0)	315(81.4)	312(85.5)	0.289	0.133
Outdoor activity(weekday)					0.500	0.002
<1 h (n, %)	114(25.3)	123(29.3)	116(30.0)	156(42.7)		
1 ~ 2 h (n, %)	222(49.3)	204(48.6)	191(49.4)	136(37.3)		
2 ~ 3 h (n, %)	68(15.1)	58(13.8)	44(11.4)	44(12.1)		
>3 h (n, %)	46(10.2)	35(8.3)	36(9.3)	29(7.9)		
Outdoor activity(weekend)					0.023	0.003
<1 h (n, %)	70(15.5)	92(21.9)	97(25.1)	132(36.2)		
1 ~ 2 h (n, %)	192(42.6)	180(42.9)	174(45.0)	140(38.4)		
2 ~ 3 h (n, %)	102(22.6)	92(21.9)	66(17.1)	41(11.2)		
>3 h (n, %)	87(19.3)	56(13.3)	50(12.9)	52(14.2)		
Sleeping time					0.046	0.720
<5 h (n, %)	2(0.4)	0(0.0)	6(1.6)	5(1.4)		
5 ~ 7 h (n, %)	18(4.0)	29(6.9)	163(42.1)	169(46.3)		
7 ~ 9 h (n, %)	326(72.3)	314(74.8)	203(52.5)	178(48.8)		
>9 h (n, %)	105(23.3)	77(18.3)	15(3.9)	13(3.6)		
Screen time(weekday)					0.292	0.010
<1 h (n, %)	241(53.6)	207(49.3)	227(58.7)	169(46.4)		
1 ~ 2 h (n, %)	135(30.0)	126(30.0)	81(20.9)	103(28.3)		
2 ~ 3 h (n, %)	46(10.2)	60(14.3)	42(10.9)	49(13.5)		
>3 h (n, %)	28(6.2)	27(6.4)	37(9.6)	43(11.8)		
Screen time(weekend)					0.279	0.711
<1 h (n, %)	90(20.0)	66(15.8)	33(8.5)	31(8.5)		
1 ~ 2 h (n, %)	182(40.4)	165(39.4)	88(22.7)	93(25.5)		
2 ~ 3 h (n, %)	101(22.4)	101(24.1)	95(24.5)	94(25.8)		
>3 h (n, %)	78(17.3)	87(20.8)	171(44.2)	147(40.3)		
Boarding school					0.336	0.342
Yes (n, %)	17(3.8)	11(2.6)	95(24.5)	79(21.6)		
Routine checking					0.884	0.442
0 time /year (n, %)	21(4.7)	16(3.9)	10(2.6)	17(4.7)		
1 time /year (n, %)	261(58.0)	246(59.4)	247(63.8)	227(62.2)		
2 time /year (n, %)	132(29.3)	116(28.0)	110(28.4)	99(27.1)		
3 time /year (n, %)	36(8.0)	36(8.7)	20(5.2)	22(6.0)		

ESG, Elementary School Graduates; JSG, Junior high School Graduates; SER, spherical equivalent refraction; P1, male vs female in ESG, P2, P1, male vs female in ESG.

### The physique indexes and the myopia

Height, weight, and body mass index between students with and without myopia were compared in Table 2. For elementary school graduates, height in males was insignificant (*p* = 0.061) in myopia but significantly higher in female myopia (*p* = 0.026), while weight and body mass index had no significant differences. For junior high school graduates, no significant differences in height, weight, and body mass index exist between myopia and emmetropic children in both male and female graduates Table 2.

**Table 2:** The comparisons of height, weight and body mass index by eyesight (mean ± SD).

Variable	Male		Female		P1	P2
	Myopia (no)	Myopia (yes)	Myopia (no)	Myopia (yes)		
ESG	*N* = 134	*N* = 317	*N* = 94	*N* = 326		
Height (cm)	157.9 ± 7.8	159.5 ± 8 0.4	156.6 ± 5.8	158.1 ± 5.7	0.06 1	0.0 26
Weight (Kg)	49.5 ± 11.5	49.1 ± 11 0.9	47.4 ± 10.4	48.3 ± 9. 5	0.71 9	0.4 53
Body mass index (Kg/m^2^)	19.7 ± 3.7	19.1 ± 3. 6	19.3 ± 3.7	19.2 ± 3. 2	0.12 7	0.9 72
JSG	*N* = 72	*N* = 315	*N* = 59	*N* = 306		
Height (cm)	171.6 ± 6.0	172.6 ± 6 0.8	162.5 ± 6.1	161.3 ± 5.3	0.27 0	0.1 01
Weight (Kg)	64.4 ± 13.2	65.0 ± 14 0.1	53.0 ± 8.4	52.8 ± 9. 1	0.70 3	0.8 71
Body mass index (Kg/m^2^)	21.8 ± 4.0	21.7 ± 4. 3	20.0 ± 2.8	20.3 ± 3. 3	0.87 8	0.5 93

P1, compared between yes and no myopia in male; P2, compared between yes and no myopia in female. ESG, Elementary School Graduates; JSG, Junior high School Graduates.

### Univariate analysis of physique indexes and SER

**Figure 1:**
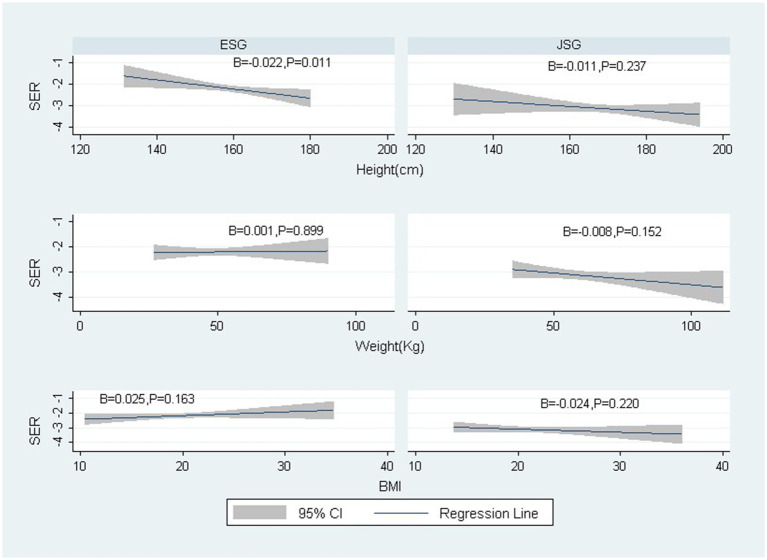
The relationships between SER (right eye) and height, weight, and Body mass index by ages. SER: spherical equivalent refraction; ESG: Elementary School Graduates; JSG: Junior high School Graduates; B: regression coefficient.

Table 3 and Figure 1 show the univariate regression analysis between physiques and SER. Height had a significantly negative relationship to SER in both eyes (*p* = 0.011, R2 = 0.006 for the right eye *p* = 0.005, R2 = 0.008 for the left eye) in elementary school graduates but not in junior high school graduates with older ages (*p* = 0.237, R2 = 0.002 for right eyes; *p* = 0.235, R2 = 0.002 for left eye).

**Table 3:** The regression coefficients of univariate analysis of physiques on SER.

Variable	Right eye			Left eye		
	B	SD	P	B	SD	P
ESG						
Height	−0.022	0.009	0.011	−0.025	0.009	0.005
Weight	0.001	0.006	0.899	−0.001	0.006	0.908
Body mass index	0.025	0.018	0.163	0.022	0.019	0.233
JSG						
Height	−0.011	0.009	0.237	−0.012	0.010	0.235
Weight	−0.008	0.006	0.152	−0.005	0.006	0.392
Body mass index	−0.024	0.019	0.220	−0.012	0.020	0.539

SER, spherical equivalent refraction; ESG, Elementary School Graduates; JSG, Junior high School. Graduate. B: regression coefficient.

### Multivariable regression analysis of height and SER

Multivariate analysis was conducted to adjust possible confounders. The results in Table 4 showed that height was still negatively and significantly related to SER on both eyes in elementary school graduates after adjusting all available confounders (*p* = 0.025 in right eye and *p* = 0.014 in left eye). Interestingly, no significant relationships between height and SER were found in older junior high school graduates (*p* = 0.886 in right eye and *p* = 0.985 in left eye).

**Table 4:** The regression coefficients of multivariate analysis of height on SER.

		Right eye			Left eye		
		B	SD	P	B	SD	P
ESG							
1	Model	−0.021	0.009	0.019	−0.024	0.009	0.009
2	Model	−0.021	0.009	0.017	−0.023	0.009	0.010
3	Model	−0.020	0.009	0.025	−0.022	0.009	0.014
JSG							
1	Model	−0.002	0.013	0.887	−0.001	0.013	0.965
2	Model	0.002	0.013	0.877	0.004	0.013	0.771
3	Model	−0.002	0.013	0.886	0.000	0.013	0.985

SER, spherical equivalent refraction; ESG, Elementary School Graduates; JSG, Junior high School Graduates. Model 1, adjusted for gender, age, and academic achievement level. Model 2, adjusted with factors in model1 plus parent’s myopia, mother education, father education, sibling numbers. Model 3, adjusted with factors in model2 plus homework time, breaks of eye using, weekday activities time, weekend activities time, weekday screentime, weekend screentime, attending boarding school.

## Discussion

This cross-sectional study was conducted in Chongqing, a municipality with a population of 34 million people in western China. Importantly, this study identified the association between spherical equivalent refraction (SER) and height in elementary school graduates. Even after accounting for available factors such as gender, age, academic performance, parental myopia, parent’s education, homework time, frequent breaks, weekday activities time, weekend activities time, weekday screentime, weekend screentime, attending boarding school, sibling, the relationship still was significant (*p* = 0.025 in right eye and *p* = 0.014 in left eye).

The findings of this study were consistent with many previous studies. For example, Saw et al. (15). studied 1,449 children aged 7–9 years and found that taller children have longer eye axis length. This negative association between height and refraction is still sustained after 5 years of follow-up (18).

A study by Huang et al., which included 65 children aged 7–9, and a serial follow-up conducted every 6 months for 3 years, it was concluded that height and AL correlated in the follow-up period (27). Chen et al. (16) found that with the increase in height, the incidence of myopia also increased in a group of 7–14 years students, which was 39.2% in the ultra-low-height group, 46.3% in the low-height group, 49.1% in the high-height group, and 58.0% in the ultra-high-height group.

Besides, Ye et al. (11) found higher heights were associated with more negative refractions (*p* < 0.05) among participants aged 6–11 years after controlling the age, gender, parental myopia, family income, reading and writing distance, and time spent outdoors. However, no association was detected between body stature and refraction from 12 to 15 years of age in Ye et al. (11) the same as in our study.

However, in this study, junior high graduates did not show an association between height and myopia or SER; the development of eyesight may explain this. In the development of eyesight, ocular dimension change is concomitant with physical development in children (28). In the early stage of life, the length of the eyes does not match the refraction produced by the cornea and lens, resulting in a defocused image on the retina. Usually, a baby is born with a natural hyperopia reserve of +2.50 to +3.00 D (29, 30). Along with the body’s height and weight growth; the eyes become longer; height is therefore positively associated with axis length (15, 31–33). While AL increases, hyperopia refractions move towards emmetropia to get a clear sight. This type of physiological change is called emmetropization. This emmetropic process will last several years and is usually finished around the age of 15 (34, 35), ending with the emmetropia refractive status of (−0.50 to +0.50 D) (35). This may be the reason to find the associations between height and myopia or SER.

Therefore, accelerated growth and the subsequent emmetropization process will quickly deplete the hyperopia reserve and increase the risk of myopia in those children. Studies showed that children with an earlier peak of their body height exhibited earlier onset myopia than those with a later peak in height or children with early peak height velocity had earlier onset of myopia than those with later peak height velocity (36).

Except for the physiological emmetropization related to physical growth, other factors such as continued near-sight work and overusing eyes can also deplete the hyperopia reserve by increasing the thickness of the cornea and lens and then causing myopia (29, 32, 35). School myopia is considered to be a consequence of multiple factors. Environmental factors, such as high educational level, the high intensity of near-sight work, and less outdoor activity, were considered significant risk factors for myopia in students (37, 38). Therefore, the loss of association between SER or myopia and height in older students may be due to the increased near-sight activities under academic pressure, which becomes the major contributor and weakens the influence of physical development on myopia.

Under China’s current education system, junior high school students experience much heavier academic pressure than elementary school students because there is competition for the Senior High School Entrance.

Heavy academic pressure made junior high students spend longer time of learning for admission to a leading senior high school, leading to more near-sight learning work and fewer outdoor activities. The increased near-sight activities not only aggravate myopia but also lead to a new onset of myopia among the students who were not myopic during the elementary school period. A comprehensive meta-analysis of the prevalence of Europeans has reported a significant relationship between higher education level and greater prevalence of myopia (39).

Some studies found body mass index associated with myopia. For example, Peled et al. (40) indicates that a high body mass index is associated with mild-to-moderate and severe myopia. Bener et al. (41) has found a significantly strong correlation between vision impairment (VA < 0.5) and body mass index.

Guan et al. (42) found that vision impairment in students was positively associated with obesity by using multiple logistic regression analysis. However, the obesity group in this study was small, with 8.3% (72/871) in elementary school graduates and 9.6% (72/752) in junior high graduates, which may explain why this study did not find an association between myopia and weight or body mass index.

### Strengths and limitations

This study has some strengths: First, the subjects included were at two distinctive physical development stages, which may help to explore the relationship between children’s physique and myopia at two age stages. Second, the measurement of SER is more accurate than the regular vision acuity chart test. Third, students were surveyed by face-to-face interviews, which may provide more precise information than a self-administrated questionnaire. Investigating the relationships between various lifestyle factors, such as academic achievement, can be used as the surrogate of the intensity of near-sight work adjusted.

However, some limitations also need to be mentioned: first, subjects came from one site, selection bias was inevitable, and the results may not be generalized to all children in another region. Second, a cross-sectional study cannot establish the causal relationship between height and myopia. Moreover, this study did not use cyclopentolate before measuring SER, which may influence the results in younger children. Also, it has not measured the axis length, which may be closer to reflecting the growth. Last, this study did not include children of other ages, which hinders the analysis of the relationship between age and myopia in this setting. Despite these limitations, the study provides valuable insight regarding the relationships between myopia and physical development in school-age students.

## Conclusion

Accelerated height growth at an early stage in children can increase the risk of myopia by accelerating the process of emmetropization. However, this relationship disappeared in junior high school graduates due to the accomplishment of emmetropization and more intensive near-sight eye use in this stage. The increased near activities should be monitored, especially in students with taller stature. A further large-scale study is warranted to investigate the relationships between growth and myopia in children of different ages.

## Data Availability

The raw data supporting the conclusions of this article will be made available by the authors, without undue reservation.
