# Supplementary material for: The association between physical stature and myopia in elementary and junior high school graduates in Chongqing, China
Source: Front Med (Lausanne). 2025 Jun 2;12:1530960. doi: 10.3389/fmed.2025.1530960 (PMC12171441; doi:10.3389/fmed.2025.1530960)
Supplement: Supplementary file 1 [file Supplementary_file_1.docx]

**Survey on Eye Health in Adolescent Students**

Dear students,

To address the national initiative on myopia prevention and control, our Health Medical Center is conducting a survey to gather relevant information regarding the occurrence of myopia. The data collected from this questionnaire will be treated with utmost confidentiality and will be used solely for statistical research analysis, without individual analysis involved. Based on the above information, if you (or the guardian of a minor) consent to participate in this survey, please kindly provide truthful responses based on your own circumstances. Thank you for your cooperation!

Name: Gender: □Male □ Female Date of Birth (MM/YY):

Primary/middle graduation school:

Middle/high school you intend to entry:

1. Are your parents nearsighted?

□Father nearsighted □Mother nearsighted

□Both nearsighted □Neither nearsighted

1. What is your mother's educational background?

□Middle school and below □High school □Junior college

□Bachelor degree □Master degree □Doctoral degree

1. What is your father's educational background?

□Middle school and below □High school □Junior college

□Bachelor degree □Master degree □Doctoral degree

1. How long do you spend on doing homework, reading, or writing after school every day (evening self-study included)?

□Less than 1 hour □1-2 hours □2-3 hours □3 hours and above

1. Do you take breaks to relax your eyes while studying or looking at electronic screens?

□Basically never □Sometimes □Often

1. At what age did you start attending extracurricular classes for cultural subjects (Chinese, mathematics, English, physics, etc.)?

□Before 3 years old □ 3-6 years old □6-9 years old □After 9 years old □Never

1. How long do you spend on extracurricular classes for cultural subjects (Chinese, mathematics, English, physics, etc.) per week?

| Primary school: | □Do not attend □ Less than 2 hours □2-4 hours |
| --- | --- |
|  | □4-8 hours □8-12 hours □More than 12 hours |
| Middle school: | □Do not attend □Less than 2 hours □2-4 hours |
|  | □4-8 hours □8-12 hours □More than 12 hours |

1. During the day on weekdays (Monday to Friday), how long do you spend on outdoor activities on average?

□Less than 1 hour □1-2 hours □2-3 hours □3 hours and above

1. During the day on weekends, holidays, and summer/winter vacations, how long do you spend on outdoor activities on average?

□Less than 1 hour □1-2 hours □2-3 hours □3 hours and above

1. How long do you sleep on average per day?

□Less than 5 hours □5-7 hours □7-9 hours □9 hours and above

1. At what age did you start using electronic devices (mobile phones, tablets, smartwatches, handheld gaming devices, etc.)?

□Kindergarten □Lower primary (Year 1-3) □Upper primary (Year 4-6)

□Middle school □Almost never

1. How long do you spend on electronic devices (mobile phones, tablets, smartwatches, etc.) per day during school days?

□Less than 1 hour □1-2 hours □2-3 hours □3 hours and above

1. How long do you spend on electronic devices (mobile phones, tablets, smartwatches, etc.) per day during weekends and holidays?

□Less than 1 hour □1-2 hours □2-3 hours □3 hours and above

1. At what age did you start wearing glasses?

□Before 6 years old □6-8 years old □9-11 years old

□12-14 years old □After 14 years old □Never

1. How often do you have your vision checked?

□Never checked □Once a year □Twice a year □3-4 times a year

1. Have you ever adopted the following measures to protect your eyes continuously for more than half a year? (Multiple choices allowed)

□Low-concentration atropine □Orthokeratology lenses □Eye-protection lamp

□Study desk and chair □Visual training equipment □None

1. Have you ever boarded during school? □Yes □No
2. Are you an only child? □Yes □No
